# Lipid–Polymeric Nanoencapsulation of Standardized *Camellia sinensis* Extract Enhances Antiangiogenic Activity and Ocular Biocompatibility

**DOI:** 10.1002/cbdv.71893

**Published:** 2026-11-01

**Authors:** Sarah Kelly Fernandes, Anízio Bastos de Souza, Líbia Andressa Leandro de Paula, Alessandra Cristine de Souza Matavel, Marcos Paulo Gomes Mol, Mariana de Oliveira Almeida, Vanessa Heloísa Ferreira de Faria, Carolina Paula de Souza Moreira

**Affiliations:** ^1^ Serviço De Desenvolvimento Tecnológico Farmacêutico, Diretoria de Pesquisa e Desenvolvimento Fundação Ezequiel Dias Belo Horizonte Minas Gerais Brazil; ^2^ Serviço De Toxinologia Molecular, Diretoria de Pesquisa e Desenvolvimento Fundação Ezequiel Dias Belo Horizonte Minas Gerais Brazil; ^3^ Serviço De Ecotoxinologia, Diretoria de Pesquisa e Desenvolvimento Fundação Ezequiel Dias Belo Horizonte Minas Gerais Brazil; ^4^ Serviço Química Especializada Diretoria Do Instituto Octávio Magalhães, Fundação Ezequiel Dias Belo Horizonte Minas Gerais Brazil

**Keywords:** antiangiogenic activity, green tea, lipid–polymeric nanoparticles, ocular biocompatibility, phytochemical characterization, polyphenols

## Abstract

*Camellia sinensis* (green tea) contains bioactive polyphenols with potential antiangiogenic activity; however, its therapeutic application is limited by extract complexity and challenges related to delivery. This study developed a lipid‐polymeric nanoparticle system containing a chemically standardized *C. sinensis* extract and evaluated its physicochemical properties, biocompatibility, and antiangiogenic activity. The optimized nanoformulation exhibited a mean diameter of 202.0 ± 5.2 nm, polydispersity index (PDI) of 0.156 ± 0.017, and zeta potential of −25.1 ± 0.6 mV, with an encapsulation efficiency of approximately 34.6 ± 1.5%. The formulation maintained suitable physicochemical characteristics during storage and showed no relevant irritation in the HET‐CAM assay. In the chick chorioallantoic membrane (CAM) assay, extract‐loaded nanoparticles reduced vascularization, displaying an antiangiogenic effect comparable to bevacizumab. These findings support lipid–polymeric nanoencapsulation as a promising strategy to improve the biological performance of complex plant‐derived extracts for ocular angiogenic disorders.

## Graphical abstract

Workflow for the synthesis, characterization, and biological evaluation of lipid–polymeric nanoparticles loaded with a chemically standardized Camellia sinensis extract, showing improved colloidal stability, ocular biocompatibility, and antiangiogenic activity.

Created by the authors using BioRender.com.

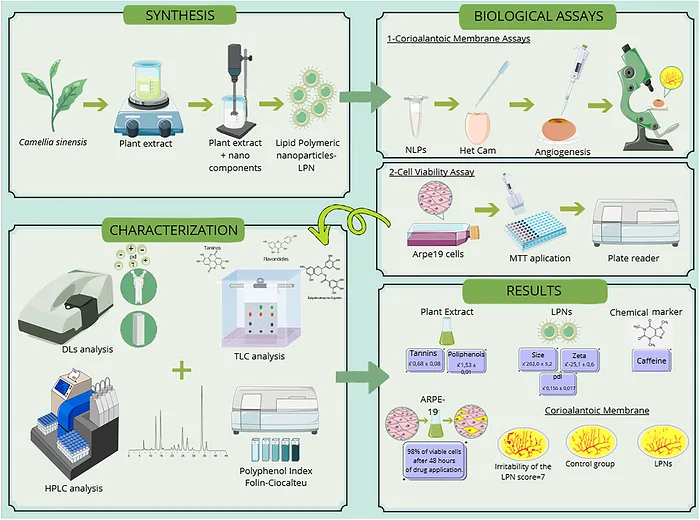

## 1 Introduction

Pathological ocular neovascularization is a hallmark of several vision‐threatening ocular diseases, including neovascular age‐related macular degeneration, diabetic retinopathy, retinal vein occlusion, and corneal neovascularization, where abnormal vessel growth disrupts tissue architecture and leads to progressive and often irreversible visual impairment [1, 2, 3, 4, 5, 6]. The clinical relevance of these diseases is amplified by their high prevalence, chronic course, and cumulative socioeconomic burden, since they require continuous monitoring, recurrent interventions, and substantial healthcare resources over extended periods [1, 2].

Currently, the treatment of ocular neovascular diseases relies primarily on intravitreal anti‐VEGF agents, including ranibizumab, aflibercept, bevacizumab, and faricimab, which have significantly improved visual outcomes in patients with neovascular retinal diseases [3, 7, 8]. However, their long‐term effectiveness remains limited by the need for repeated intravitreal injections, variable therapeutic response, treatment burden, and recurrence after therapy discontinuation, underscoring the need for alternative or adjunctive strategies with improved durability and broader mechanisms of action [3, 9].

Despite the remarkable pharmacological potential of natural products, their successful ophthalmic application remains limited by the unique anatomical and physiological barriers of the eye. Tear turnover, blinking, nasolacrimal drainage, and the multilayered corneal epithelium rapidly eliminate topically administered formulations, allowing typically less than 5% of the instilled dose to reach intraocular tissues and resulting in poor ocular bioavailability and the need for frequent dosing [10, 11, 12]. These challenges highlight the importance of developing advanced ophthalmic drug delivery systems capable of prolonging precorneal residence time, enhancing ocular bioavailability, protecting labile compounds from degradation, and providing sustained drug release [12]. In this context, ophthalmic research has shown increasing interest in natural products and nutraceuticals as sources of bioactive compounds with antioxidant, anti‐inflammatory, antiapoptotic, and antiangiogenic properties, reflecting their growing potential as complementary therapeutic strategies for ocular diseases [8, 13, 14, 15].

Among medicinal plants, *Camellia sinensis* (green tea) has emerged as one of the most extensively investigated botanical sources owing to its high content of catechins and related polyphenols, including epigallocatechin‐3‐gallate, epigallocatechin, epicatechin gallate, and epicatechin, whose composition depends on cultivar, processing, and extraction conditions [16, 17, 18, 19]. These compounds exhibit antioxidant, anti‐inflammatory, and antiangiogenic activities through modulation of endothelial proliferation, migration, oxidative stress, and VEGF‐mediated signaling pathways [18, 19, 20]. Nevertheless, the clinical translation of *Camellia*‐derived extracts remains challenging due to its chemical instability, variability in phytochemical composition, and limited ocular bioavailability, making extract standardization and optimized delivery systems essential for maximizing their pharmacological potential [21, 22, 23]. Since the pharmacological activity of botanical extracts is directly influenced by their phytochemical composition, chemical standardization represents a critical prerequisite for ensuring batch‐to‐batch consistency, reproducible biological responses, and the successful translation of plant‐derived products into clinically relevant ophthalmic formulations [24, 25, 26].

Nanotechnology‐based delivery systems have therefore emerged as promising platforms for improving the ophthalmic administration of phytochemicals [13]. By protecting labile compounds from degradation, enhancing solubility, prolonging precorneal retention, and enabling controlled drug release, nanoparticles can substantially improve the biological performance of natural products [27, 28]. Compared with conventional polymeric or lipid nanoparticles, hybrid lipid–polymeric nanoparticles integrate the advantages of both systems, offering improved structural stability, controlled drug release, high encapsulation efficiency, and excellent ocular biocompatibility [29, 30]. This hybrid architecture represents a particularly attractive strategy for topical ocular administration because it can improve the stability and therapeutic performance of polyphenol‐rich extracts while maintaining physicochemical characteristics suitable for ocular application [30, 31]. Because topical ophthalmic formulations come into direct contact with sensitive ocular tissues, the preliminary assessment of ocular irritation is a critical step during preclinical development. In this context, the Hen's Egg Test–Chorioallantoic Membrane (HET‐CAM) has been widely adopted as an alternative method for the early evaluation of ocular irritation potential, providing a rapid, reliable, and ethically acceptable screening tool for the preliminary safety assessment of ophthalmic formulations before in vivo studies [32, 33].

Therefore, the present study aimed to chemically standardize an aqueous extract of *Camellia sinensis*, incorporate the standardized extract into lipid–polymeric nanoparticles, characterize the resulting nanosystem, and investigate whether nanoencapsulation improves the antiangiogenic efficacy and ocular biocompatibility of the standardized extract. Furthermore, establishing relationships between the phytochemical composition of standardized extracts and their biological performance is essential for the rational development of reproducible phytopharmaceutical formulations.

## 2 Results and Discussion

### 2.1 Phytochemical Characterization and Quality Assessment of *Camellia sinensis* Extract

The extraction of dried *Camellia sinensis* leaves yielded 19.2% (w/w) of lyophilized aqueous extract (GTE), consistent with values reported for green tea preparations obtained by decoction [34, 35]. The resulting material appeared as a fine green‐brown powder and was stored under controlled conditions to prevent oxidative degradation of phenolic constituents.

Qualitative phytochemical screening by thin‐layer chromatography (TLC), using literature‐established mobile phases and visualization reagents [36] indicated the presence of phenolic compounds, including catechins/tannins and flavonoids, based on their characteristic color reactions following derivatization with specific visualization reagents. The results of the qualitative phytochemical screening are summarized in Table 1. These findings are consistent with the known phytochemical profile of green tea, in which catechins may account for up to 70% of total polyphenols and represent 30%–42% of leaf dry weight [34, 35]. The strong detection of tannins and flavonoids reinforces the chemical identity of the extract and supports its classification as a polyphenol‐rich matrix.

**TABLE 1:** Qualitative identification of major classes of secondary metabolites in the aqueous extract of *Camellia sinensis* (GTE).

Phytochemical group	Eluent (proportion)	Standard used	Visualization reagent	Detection
Terpenes	hexane/ethyl acetate (90:10)	β‐sitosterol	Vanillin/H_2_SO_4_	—
Tannins/Catechins	ethyl acetate/formic acid/ water (120:10:10)	Pyrogallol + gallic acid	Ferric chloride	+++
Flavonoids	ethyl acetate/formic acid/ acetic acid/ water (100:11:11:27)	Rutin + quercetin	(NP/PEG)—UV_365_ nm	+++
Cumarins	hexane/dichloromethane/ acetone (45:25:30)	Cumarin	KOH 5%—UV_365_ nm	++
Saponins	ethyl acetate/formic acid/ acetic acid/ water (100:11:11:27)	Asiaticoside	Vanillin/H_2_SO_4_	—

Abbreviations: −, absence; NP/PEG, Natural Product Reagent/ Polyethylene glycol; ++, moderate presence; +++, high presence.

Besides catechins, *C. sinensis* contains a complex mixture of bioactive phytochemicals whose composition is strongly influenced by cultivar, geographic origin, agronomic conditions, leaf maturity, processing, and extraction procedures. Consequently, the biological activity of green tea extracts depends not only on the presence of individual compounds but also on their relative proportions and possible synergistic interactions within the phytochemical matrix [24, 26, 37]. The major catechins reported in *C. sinensis* include epigallocatechin‐3‐gallate (EGCG), generally the predominant compound, followed by epigallocatechin (EGC), epicatechin gallate (ECG), and epicatechin (EC). These flavan‐3‐ols possess multiple hydroxyl groups that confer high antioxidant capacity and contribute to the modulation of oxidative stress, inflammatory signaling, and angiogenic pathways. However, considerable variability in catechin and total polyphenol contents has been reported among commercial green tea products as a consequence of differences in cultivar, geographical origin, agronomic practices, leaf processing, and extraction procedures, reinforcing the importance of chemical standardization and quality control to ensure reproducibility and consistency of plant‐derived raw materials [35, 37].

The total polyphenol and tannin contents of GTE were quantified by spectrophotometric methods [38], and the results are summarized in Table 2. Tannins represented approximately 44% of the total Folin‐reactive phenolic compounds, indicating that they constitute an important fraction of the phenolic constituents present in the extract. This finding is consistent with the characteristic phytochemical composition of green tea, which is rich in tannins and other phenolic compounds, including catechins. Similar ranges of total phenolics have been reported for commercial *Camellia sinensis* preparations, although their composition is influenced by cultivar, processing conditions, extraction procedure, and analytical methodology [34, 39]. The high phenolic content reinforces the chemical profile observed in the qualitative screening and supports the extract standardization strategy adopted in the present study.

**TABLE 2:** Quantitative phenolic profile of green tea extract (GTE) determined by spectrophotometric assays.

Analyte	Content*
Total polyphenols	1.53 ± 0.01 mg GAE/mL**
Total tannis	0.68 ± 0.03 mg GAE/mL**

* Values represent mean ± standard deviation (*n* = 3).

** GAE, gallic acid equivalents.

The high phenolic content is particularly relevant for the subsequent development of the nanoformulation. Polyphenols, especially catechins are highly susceptible to oxidation and degradation during processing and storage, which may compromise their biological activity. Therefore, the incorporation of GTE into polymeric nanoparticles represents a rational strategy to protect these bioactive compounds, improve their physicochemical stability, and promote a more sustained release profile.

Reversed‐phase high‐performance liquid chromatography (RP‐HPLC) further complemented the chemical characterization of the extract by providing the selective identification and quantification of individual marker compounds. Caffeine and rutin were identified by comparing their retention times and UV spectra with those of authenticated reference standards (Figure 1). Among these compounds, caffeine was selected as the quantitative marker because of its chemical stability, consistent occurrence in *C. sinensis*, and excellent chromatographic performance. Quantification by external calibration yielded a caffeine concentration of 61.96 ± 2.13 µg/mL, corresponding to 3.10 ± 0.13% (w/w) of the dry extract. This value is consistent with the caffeine content generally reported for green tea leaves and aqueous extracts, which typically ranges from 2% to 5% of the dry weight [35, 37, 40]. The observed caffeine level therefore confirms the authenticity and chemical quality of the extract while supporting the suitability of caffeine as a quantitative marker for quality control. The well‐resolved chromatographic peak and the excellent linearity of the calibration curve (R^2^ = 0.9998) demonstrate the robustness and reliability of the RP‐HPLC method for caffeine determination. Unlike the Folin–Ciocalteu assay, which estimates the overall content of reducing phenolic compounds, RP‐HPLC coupled with UV detection provides high analytical specificity for individual constituents. Therefore, the combined use of spectrophotometric assays for total polyphenols and tannins together with chromatographic quantification of caffeine constitutes a robust strategy for the chemical standardization and quality control of the green tea extract [34, 35, 38].

**FIGURE 1:**
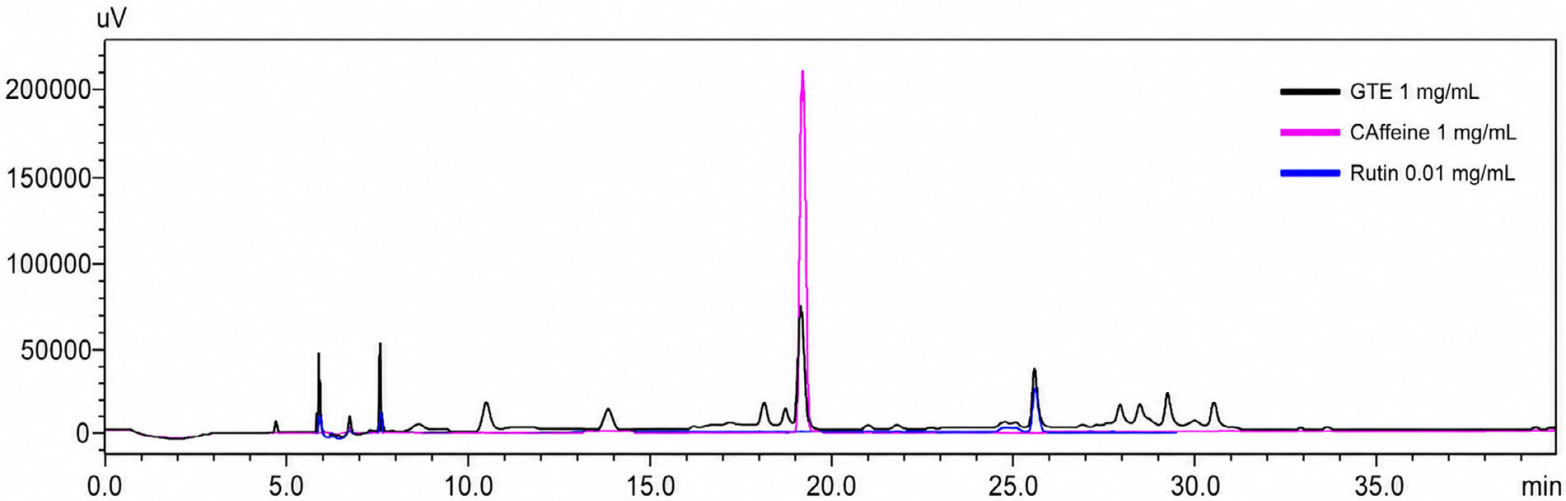
RP‐HPLC chromatograms of green tea extract (GTE) and reference standards (caffeine and rutin).

In the present study, TLC screening, spectrophotometric assays, and HPLC analyses consistently demonstrated the occurrence of phenolic constituents, particularly catechins and other polyphenols, supporting the chemical profile of the standardized extract. Whereas catechins are recognized as the main contributors to the antioxidant and antiangiogenic activities of green tea, caffeine may also modulate cellular responses and has been reported to exert antiangiogenic effects by influencing endothelial cell proliferation and vascular signaling pathways. The quantitative determination of total phenolics, tannins, and caffeine further strengthens the phytochemical characterization, providing complementary evidence that cannot be inferred from TLC alone. Such chemical standardization is an essential prerequisite for ensuring batch‐to‐batch consistency and reproducible pharmacological responses, thereby supporting the reliable interpretation of the biological activities observed for the developed formulation [26, 37].

The phytochemical profiling and definition of chemical markers are essential steps for establishing the identity and consistency of green tea raw material. However, according to the Brazilian Pharmacopoeia (seventh ed.), quality control of vegetal drugs must also include the evaluation of pesticide residues to ensure safety and suitability for pharmaceutical use [41]. In this context, the combined assessment of chemical markers and residual agrochemicals provides a comprehensive framework for raw material qualification, strengthening the reliability of subsequent formulation, encapsulation, and biological studies, as s recommended by major pharmacopoeias and international guidelines.

The proposed analytical method was validated for the determination of 205 pesticide residues, comprising 176 compounds analyzed by liquid chromatography–tandem mass spectrometry (LC–MS/MS) and 37 compounds analyzed by gas chromatography–tandem mass spectrometry (GC–MS/MS) [42]. For LC–MS/MS analyses, limits of detection (LOD) and quantification (LOQ) ranged from 0.0001 to 0.0004 mg/kg and from 0.0002 to 0.0008 mg/kg, respectively, whereas for GC–MS/MS analyses, LOD and LOQ values ranged from 0.001 to 0.004 mg/kg and from 0.002 to 0.008 mg/kg. Among all screened compounds, only carbendazim was detected, and its concentration was below the LOQ, while all other pesticides were below the LOD, indicating negligible contamination.

Large‐scale surveys of plant‐derived foods have reported frequent detection of pesticide residues, with approximately 30% of vegetable samples containing measurable levels and a subset exceeding established maximum residue limits, particularly in leafy crops [43]. Within this context, the absence of quantifiable residues in the analyzed *C. sinensis* material confirms its compliance with current Brazilian regulatory requirements for herbal raw materials. The integration of extensive pesticide screening with phytochemical standardization reinforces the analytical robustness of the study and supports the suitability of the selected raw material for subsequent nanoformulation and biological evaluation.

Having established the chemical identity, quantitative marker profile, and compliance with pesticide residue regulations, the standardized *C. sinensis* extract was considered suitable for technological development. The incorporation of chemically characterized plant extracts into delivery systems represents a rational strategy to enhance their functional performance while preserving compositional integrity. In this context, the development and physicochemical evaluation of lipid–polymeric nanoparticles loaded with the standardized extract were subsequently undertaken.

### 2.2 Development and Physicochemical Characterization of the Lipid–Polymeric Nanoparticles

Lipid–polymeric nanoparticles were prepared by nanoprecipitation using poly(ε‐caprolactone) (PCL) as the polymeric matrix and phosphatidylcholine as the lipid component, stabilized with nonionic surfactants (Table 3). The hydrophobic and biodegradable properties of PCL favor the incorporation of poorly water‐soluble constituents and may contribute to controlled release behavior [44, 45].

**TABLE 3:** Composition of green tea extract (GTE)‐loaded lipid–Polymeric nanoparticles.

Component *	Content (% w/w)
GTE	0.7
PCL	34.8
PC	15.7
PS20	7.0
P188	38.3

Abbreviations: GTE, green tea extract; P188, poloxamer 188; PC, phosphatidylcholine; PCL, polycaprolactone; PS20, polysorbate 20.

Phosphatidylcholine contributes to interfacial organization and colloidal stabilization in aqueous media, while polysorbate 20 reduces interfacial tension during nanoparticle formation. Poloxamer 188 provides steric stabilization and has been associated with improved tolerability in ophthalmic systems [44, 46, 47].

The resulting suspension presented a homogeneous milky appearance. The physicochemical parameters of blank and GTE‐loaded nanoparticles are summarized in Table 4. Both systems exhibited nanoscale hydrodynamic diameters and low polydispersity indices (PdI < 0.2), indicating narrow size distribution and colloidal uniformity. The incorporation of GTE resulted in a slight reduction in hydrodynamic diameter compared to blank nanoparticles, a behavior also reported for other plant extract‐loaded polymeric systems.

**TABLE 4:** Physicochemical parameters of blank and GTE‐loaded nanoparticles.

Formulation	Hydrodynamic diameter (nm)	Zeta potential (mV)	PdI	Encapsulation efficiency (%)
GTE‐loaded	202.0 ± 5.2	−25.1 ± 0.6	0.156 ± 0.017	34.6 ± 1.5
Blank	255.1 ± 26.4	−30.7 ± 8.9	0.153 ± 0.046	NA

Abbreviations: GTE, green tea extract; *n* = 3; NA, not applicable. Data are presented as mean ± SD; PdI, polydispersity index.

Negatively charged surface potentials (approximately −25 mV) were observed for both formulations, suggesting electrostatic contribution to colloidal stability [30]. The encapsulation efficiency of the extract was 34.6 ± 0.5%, indicating partial incorporation of the phytochemical constituents into the polymeric matrix.

The physical stability of both formulations was monitored over a three‐month period by assessing hydrodynamic diameter, zeta potential, and polydispersity index (PdI). Blank nanoparticles exhibited limited variation, while GTE‐loaded nanoparticles maintained consistent physicochemical profiles (Figure 2). These parameters remained within narrow intervals throughout the monitoring period, indicating maintenance of colloidal stability without evidence of significant aggregation or broadening of the size distribution.

**FIGURE 2:**
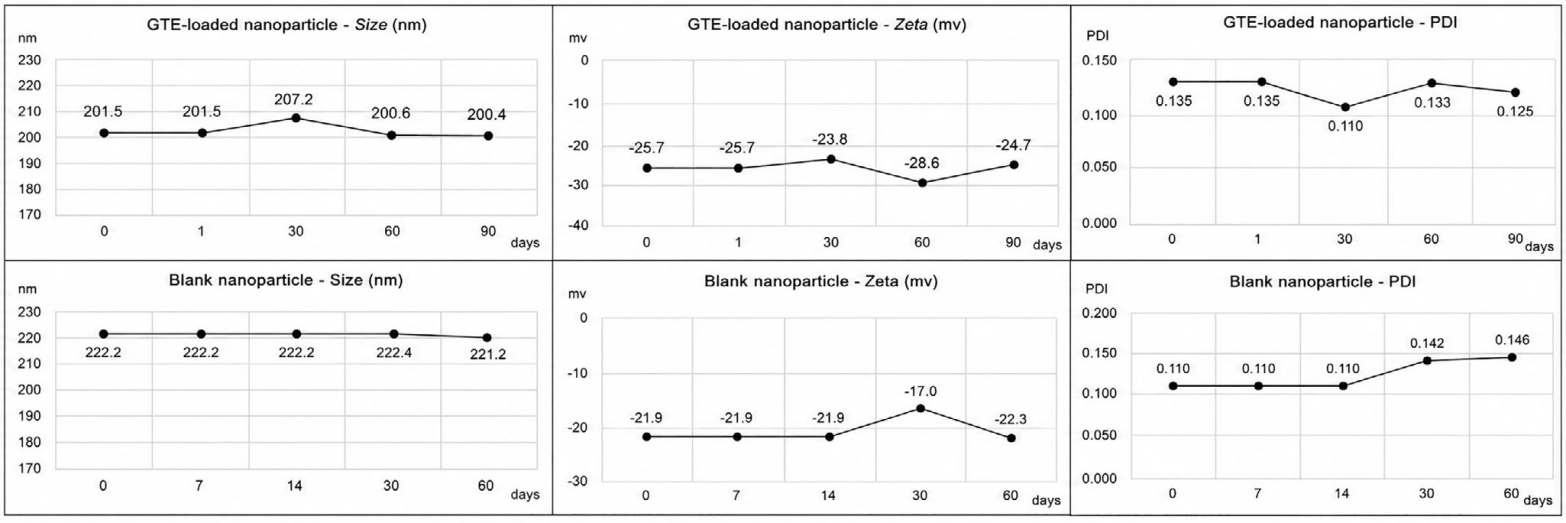
Stability profiles of blank and GTE‐loaded nanoparticles over three months (mean, *n* = 3).

The physical stability of polymeric nanosystems is a key attribute reflecting their ability to preserve colloidal integrity under storage conditions relevant to downstream applications. Poly(ε‐caprolactone) (PCL) has been widely investigated as a biodegradable polymer for nanoparticle formulation due to its favorable properties including slow degradation rate, biocompatibility, and relatively high physical stability compared with other polyester polymers. For example, comparative stability studies of biodegradable polymeric nanoparticles demonstrated that PCL nanoparticles exhibited greater stability during storage at both room temperature and 4°C when compared to certain poly(lactic‐co‐glycolic acid) systems, with minimal changes in particle size over prolonged periods [31]. For instance, PCL nanoparticles encapsulating plant bioactives have retained narrow size distributions (PdI < 0.2) and stable surface charge in storage stability assessments, which is generally interpreted as an indicator of robust colloidal behavior suitable for further biological evaluations [48].

The stability of colloidal carriers has direct implications for their performance in biological assays. Stable particle size distributions ensure reproducible biodistribution profiles and consistent interactions at the cell surface, which in turn affect cellular uptake and downstream biological responses [49]. Moreover, surface charge plays a role in modulating protein corona formation and cellular internalization pathways, with negatively charged particles frequently showing reduced nonspecific adsorption and lower cytotoxicity when compared to highly cationic counterparts [50]. In the context of the present study, the observed stability of GTE‐loaded nanoparticles supports their use in subsequent biological evaluations, such as antiangiogenic assays.

The RP‐HPLC method was also applied to determine the encapsulation efficiency of the prepared nanoparticles, as described in a subsequent section. The encapsulation efficiency (EE) of the nanoparticles, determined by RP‐HPLC quantification of non‐encapsulated caffeine, was 34.6 ± 1,5%. The moderate encapsulation efficiency observed may be related to the complex and chemically diverse composition of *C. sinensis* extract, which contains polyphenols with different molecular characteristics and affinities for the lipid–polymeric matrix. This result is consistent with the physicochemical properties of caffeine, a small and highly hydrophilic molecule (logP ≈ −0.1), which exhibits limited affinity for the hydrophobic poly(ε‐caprolactone) (PCL) matrix. During nanoparticle formation by the emulsification–solvent evaporation method, hydrophilic compounds tend to partition preferentially into the aqueous phase, reducing their incorporation into the polymeric core [30, 48, 51]. Consequently, moderate encapsulation efficiencies are generally expected for hydrophilic molecules in PCL‐based nanoparticles, whereas considerably higher EE values are commonly achieved for lipophilic compounds owing to their greater affinity for the polymer matrix. Therefore, the EE observed in the present study is compatible with the physicochemical characteristics of caffeine and confirms that the developed nanosystem was able to incorporate a representative fraction of this hydrophilic marker while maintaining satisfactory formulation performance.

From a mechanistic perspective, PCL forms a predominantly hydrophobic polymeric core that preferentially accommodates less polar constituents, whereas more hydrophilic fractions remain partially unassociated. The inclusion of phosphatidylcholine may facilitate interfacial stabilization of amphiphilic molecules; however, it does not fully prevent partitioning into the aqueous medium during solvent displacement [52].

Importantly, the presence of a partially unencapsulated fraction may influence the initial interaction of the formulation with biological tissues. Therefore, evaluating the ocular safety profile and antiangiogenic activity of both free and nanoencapsulated GTE becomes essential to determine whether nanoencapsulation effectively modulates tissue exposure and biological response. In this context, cytotoxicity, irritation, and angiogenesis assays were conducted to elucidate the functional implications of the physicochemical properties described above.

### 2.3 Biological Evaluation: Ocular Safety and Antiangiogenic Activity

To investigate the in vitro effect of GTE on toxicity in a human retinal pigment epithelial cell line, a cell‐viability assay using ARPE‐19, a well‐established model, was used to test its safety. The rationale for using this cell line in toxicological studies is that the once administered to the posterior portion of the eye, the formulation may penetrate through the retinal layers and cause toxicity to the RPEs [53].

GTE exhibited a concentration‐dependent but time‐independent cytotoxic effect (*p* > 0.05), with mean IC_50_ values of 0.22 ± 0.042 mg·mL^−^^1^ at 24 h, 0.21 ± 0.045 mg·mL^−^^1^ at 48 h, and 0.23 ± 0.052 mg·mL^−^^1^ at 72 h (Figure 3). The consistency of IC_50_ values across exposure times indicates the absence of cumulative cytotoxic stress within the tested interval. These results suggest that cellular toxicity is primarily concentration‐driven rather than progressively time‐dependent.

**FIGURE 3:**
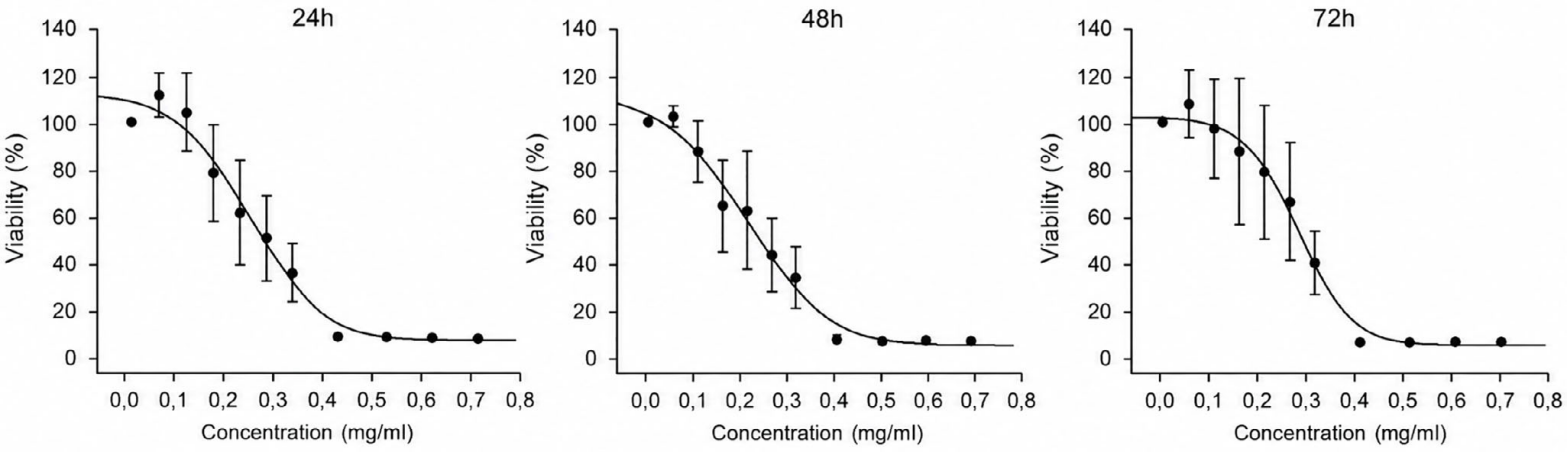
Dose–response curves of GTE in ARPE‐19 cells after 24, 48, and 72 h of exposure. Error bars represent standard deviation (SD). ARPE‐19 cells were exposed to GTE at concentrations ranging from 0.05 to 0.7 mg·mL^−^^1^ for 24, 48, and 72 h. Cell viability was determined using a dose–response analysis, and IC_50_ values were calculated using a four‐parameter logistic regression model. The obtained IC_50_ values were 0.22 ± 0.042 mg·mL^−^^1^ (24 h, *n* = 5), 0.21 ± 0.045 mg·mL^−^^1^ (48 h, *n* = 5), and 0.23 ± 0.052 mg·mL^−^^1^ (72 h, *n* = 4).

Importantly, the concentrations employed in nanoparticle formulation remain within a range compatible with cellular viability, providing a safety margin for ocular application. Although high concentrations of polyphenols have been associated with oxidative stress in retinal models [49], the stability of the IC_50_ values observed here indicates a controlled cytotoxic profile under the experimental conditions.

While in vitro cytotoxicity assays provide insight into epithelial compatibility, the Hen's Egg Test on the Chorioallantoic Membrane (HET‐CAM) allows evaluation of vascular and irritative responses in a complex biological environment. GTE at 0.2 mg·mL^−^^1^ produced a high irritation score (IS = 9.8), corresponding to the threshold of severe irritancy according to standard classification criteria [33]. This response is consistent with the concentration‐dependent epithelial sensitivity observed in ARPE‐19 cells and may be attributed to the high phenolic content of the extract, which can interact directly with vascular endothelium and epithelial membranes.

Encapsulation significantly attenuated this response. GTE‐loaded nanoparticles exhibited a lower irritation index (IS = 7.0), classified as moderately irritant, indicating that nanoencapsulation mitigates direct tissue exposure to free phytochemicals. Blank nanoparticles showed only slight irritation (IS = 1.8), supporting the intrinsic biocompatibility of the lipid–polymeric matrix composed of poly(ε‐caprolactone) and phosphatidylcholine.

These findings indicate that the nanocarrier system modulates the biological interface by controlling extract release and reducing immediate vascular insult. The residual irritation observed for GTE‐loaded nanoparticles may be related to the moderate encapsulation efficiency, suggesting the presence of a free extract fraction contributing to the irritative response.

Together, the concordant ARPE‐19 and HET‐CAM results (Figure 4) demonstrate that nanoencapsulation improves ocular tolerability compared to the free extract while preserving biological activity.

**FIGURE 4:**
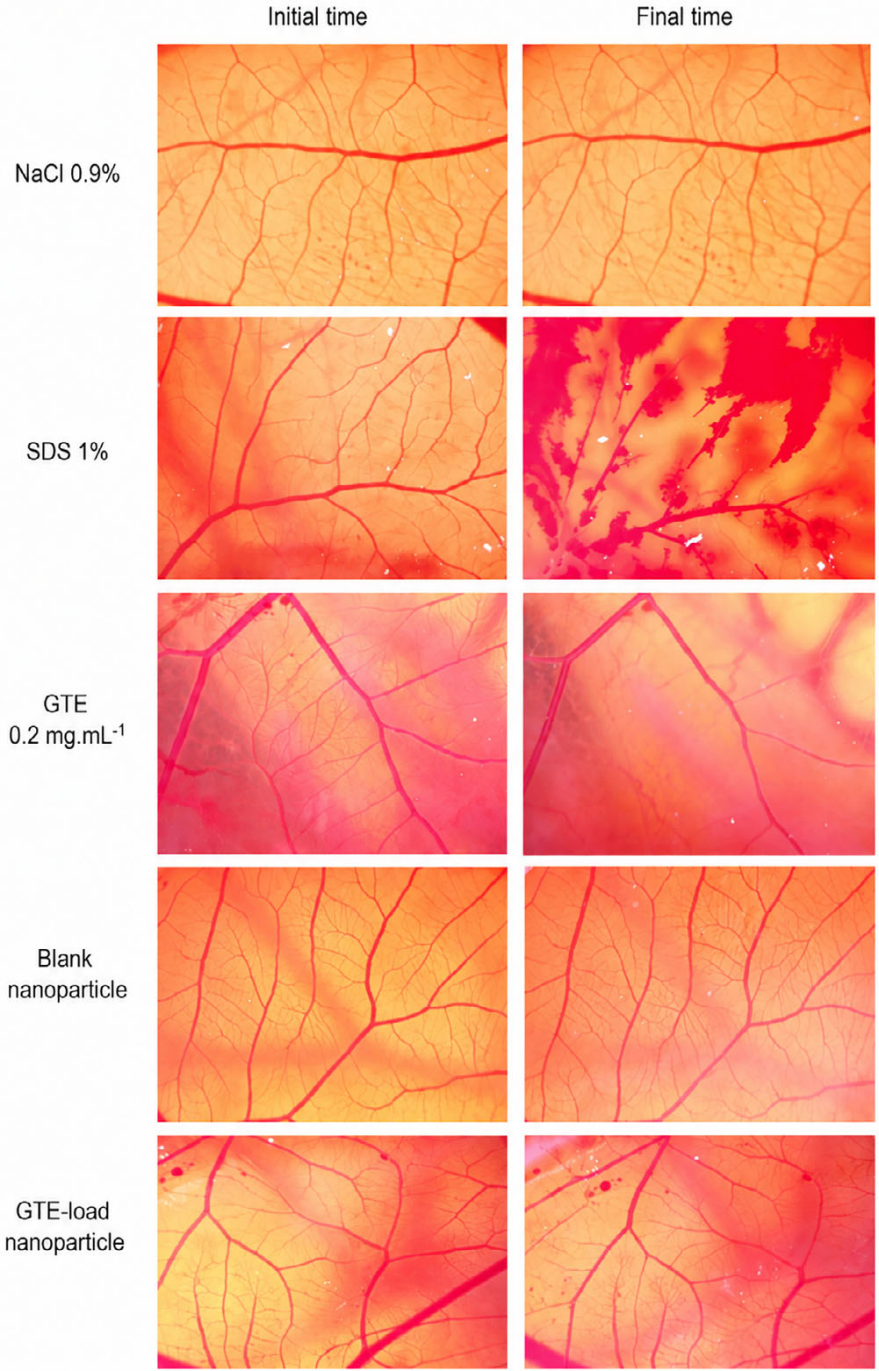
Representative images of vascular responses in the HET‐CAM assay after exposure to the tested formulations. Representative images of vascular alterations (hemorrhage, lysis, and coagulation) observed in the HET‐CAM assay after treatment with the indicated formulations. Images were captured during the observation period (5 min) used for irritation score calculation. NaCl 0.9% is negative control; SDS, sodium dodecyl sulfate is positive control; GTE, green tea extract.

The chorioallantoic membrane (CAM) assay was employed to investigate the antiangiogenic potential of the developed system. Green tea catechins, particularly epigallocatechin‐3‐gallate (EGCG) have been widely reported to inhibit angiogenesis through modulation of multiple signaling pathways, including vascular endothelial growth factor (VEGF), cyclooxygenase‐2 (COX‐2), matrix metalloproteinases, interleukin‐1β (IL‐1β), and the nuclear factor‐κB (NF‐κB) cascade [18]. The preservation of phenolic constituents through nanoencapsulation is expected to maximize their biological availability at the target site, thereby enhancing the inhibition of neovascularization while minimizing degradation of the active compounds.

In the present study, both free GTE and GTE‐loaded nanoparticles significantly reduced neovascularization (Figure 5). GTE reduced vascularization by 36.38 ± 4.51%, whereas GTE‐loaded nanoparticles achieved 41.61 ± 4.82% inhibition, approaching the effect observed for Bevacizumab (42.35 ± 5.09%), a clinically used anti‐VEGF monoclonal antibody. Nonparametric multiple comparisons using the Kruskal–Wallis test followed by Dunn's post hoc analysis revealed statistically significant differences among groups (*p* < 0.001). As expected, the PBS‐treated group (negative control) differed significantly from all treatment groups. Although bevacizumab exhibited the highest inhibition values, the magnitude of response observed for GTE‐loaded nanoparticles was biologically comparable, indicating relevant antiangiogenic potential.

**FIGURE 5:**
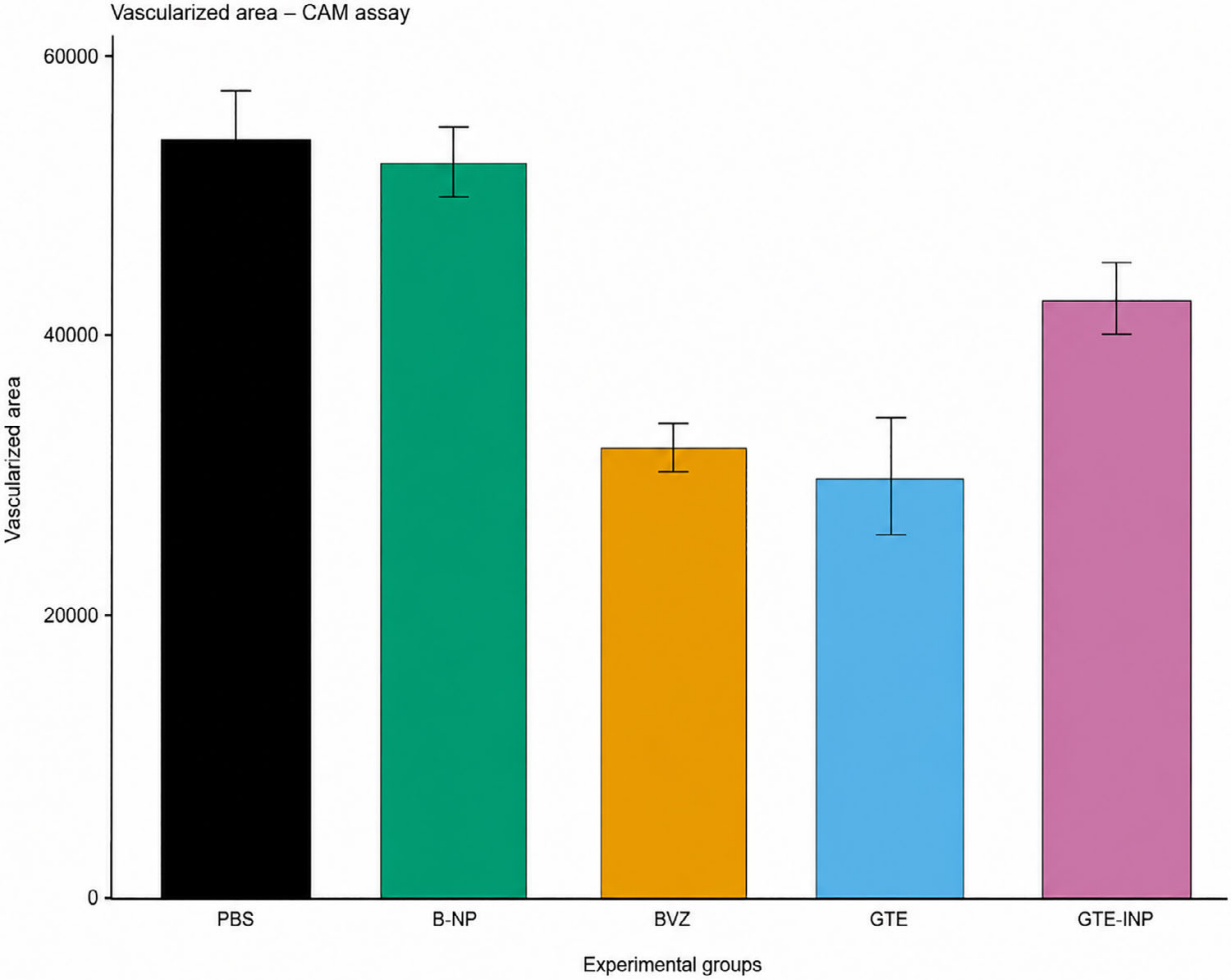
Quantitative analysis of neovascularization in the CAM assay across experimental groups. PBS, Phosphate‐Buffered Saline (Negative control); BNP, Blank nanoparticle; BVZ, Bevacizumabe (Positive control); GTE, green tea extract; GTE‐NP, green tea‐loaded nanoparticle. Error bars represent standard deviation (SD); *n* = 10. Statistical analysis was performed using the Kruskal–Wallis test followed by Dunn's multiple comparisons test. Significant differences (95% confidence level) were observed between PBS and BVZ (*p* = 0.0000418), PBS and GTE (*p* = 0.0000170), B‐NP and BVZ (*p* = 0.000115), and B‐NP and GTE (*p* = 0.0000485).

The enhanced inhibitory effect observed after nanoencapsulation may reflect improved local bioavailability and sustained release of active catechins at the vascular interface. Given the known instability and limited bioavailability of phenolic compounds, nanoformulation appears to enhance therapeutic performance by stabilizing the extract and modulating its tissue interaction profile.

Importantly, the suppression of neovascularization cannot be attributed solely to nonspecific cytotoxicity, as cellular viability assays demonstrated concentration‐dependent but non‐progressive toxicity. Instead, the data support a mechanism involving biochemical modulation of angiogenic signaling pathways, consistent with documented VEGF pathway interference by green tea catechins.

Although the present study did not directly evaluate angiogenesis‐related molecular markers, the observed phenotypic inhibition of vascularization is consistent with the known biological activity of polyphenols from *C. sinensis*. In the chicken chorioallantoic membrane (CAM) assay, green tea catechins have been previously shown to significantly reduce angiogenesis induced by pro‐angiogenic stimuli, demonstrating a direct inhibitory effect on vascular development in vivo [18, 50].

Mechanistically, catechins particularly epigallocatechin‐3‐gallate (EGCG) are known to modulate key angiogenic pathways, including the vascular endothelial growth factor signaling axis, leading to inhibition of endothelial cell proliferation and capillary formation [54]. These mechanisms support the antiangiogenic response observed in the present study and reinforce the relevance of green tea polyphenols as modulators of vascular growth.

The present study has some limitations that should be acknowledged. Although the present findings demonstrate the physicochemical suitability, preliminary ocular safety, and antiangiogenic potential of the developed lipid–polymeric nanoparticles, further investigations including long‐term stability studies, ocular pharmacokinetics, and in vivo efficacy and safety evaluations are warranted to support their translational development and potential clinical application.

## 3 Conclusions

This study demonstrates that lipid–polymeric nanoencapsulation of a chemically standardized extract from *Camellia sinensis* enhances its antiangiogenic performance while improving biocompatibility in ocular‐relevant experimental models. By stabilizing and modulating the delivery of green tea polyphenols, the nanocarrier system enables controlled biological interaction with the vascular interface, resulting in inhibition of angiogenic responses without inducing pronounced irritation. In the CAM assay, the inhibition of vascularization achieved by the nanoformulation approached that observed for bevacizumab, highlighting the potential of multitarget, plant‐derived nanotherapeutics to modulate angiogenic processes through mechanisms distinct from those of single‐target biologics. Collectively, these findings support nanoformulation as a chemical biology strategy to harness the therapeutic potential of complex natural products and provide a foundation for the development of alternative approaches for the management of ocular angiogenic disorders.

Despite these promising findings, this study should be considered a proof‐of‐concept demonstrating the feasibility of using a lipid–polymeric nanoplatform to enhance the biological performance and biocompatibility of a chemically standardized *C. sinensis* extract. The results provide an important foundation for the further development of plant‐derived nanotherapeutics; however, several limitations should be acknowledged. The study was performed using preclinical in vitro and ex vivo models, which do not fully reproduce the complexity of the ocular environment, including physiological barriers, tear dynamics, and in vivo pharmacokinetic behavior. In addition, the encapsulation efficiency was moderate, and the molecular mechanisms underlying the antiangiogenic activity were not directly investigated. Future studies should focus on optimizing formulation performance and encapsulation efficiency, evaluating long‐term physicochemical stability, and investigating ocular pharmacokinetics, biodistribution, efficacy, and safety in relevant animal models. Further elucidation of the signaling pathways involved in the antiangiogenic response will also be essential to clarify the mechanism of action and facilitate the translation of this nanoplatform toward potential clinical applications.

## 4 Experimental Section

### 4.1 Chemicals and Reagents

Dried leaves were commercially supplied and labeled as *Camellia sinensis* (A Floresta, Belo Horizonte, Brazil, lot 102902052022). Since the plant material was not collected by the authors, no voucher specimen was deposited. Poly(ε‐caprolactone) (PCL), polysorbate 20 (Tween 20), poloxamer 188 (Kolliphor P188), caffeine, gallic acid, rutin, epigallocatechin‐3‐gallate (EGCG), ferric chloride, aluminum chloride, Folin–Ciocalteu reagent, sodium carbonate, potassium hydroxide, and dimethyl sulfoxide (DMSO) were purchased from Sigma‐Aldrich (St. Louis, MO, USA). Phosphatidylcholine (Lipoid S100) was obtained from Lipoid GmbH (Ludwigshafen, Germany). HPLC‐grade methanol, acetonitrile, dichloromethane, acetone, hexane, ethyl acetate, acetic acid, and formic acid were supplied by Merck (Darmstadt, Germany). Silica gel 60 F_254_ thin‐layer chromatography plates were purchased from Merck (Darmstadt, Germany).

Human retinal pigment epithelial cells (ARPE‐19; ATCC CRL‐2302, RRID: CVCL_0145) were obtained from the American Type Culture Collection (ATCC, Manassas, VA, USA) and cultured in DMEM/F12 medium supplemented with fetal bovine serum (FBS; Gibco, Thermo Fisher Scientific). Phosphate‐buffered saline (PBS) was obtained from Sigma‐Aldrich. Bevacizumab (Avastin) was obtained from Genentech/Roche (South San Francisco, CA, USA). Fertilized *Gallus gallus domesticus* eggs were supplied by a certified hatchery (Brazil).

### 4.2 Preparation of Green Tea Extract (GTE)

Dried leaves of *Camellia sinensis* were manually inspected to remove foreign material and ground to obtain a homogeneous powder. The aqueous extract was prepared by decoction of powdered leaves (50 g) in distilled water (300 mL) for 30 min. After cooling to room temperature, the extract was filtered through cotton and lyophilized. The dried extract (GTE) was stored in a desiccator protected from light and moisture until further use.

### 4.3 Phytochemical Screening

Preliminary phytochemical analysis of GTE was performed by thin‐layer chromatography (TLC). Samples (1 mg·mL^−^^1^) and reference standards were applied to silica gel 60 F254 plates and developed in pre‐saturated chambers using appropriate mobile phases reported in the literature [36]. The complete chromatographic systems, including mobile phases, visualization reagents, and reference standards, are presented in Table 1. After development, plates were air‐dried and visualized under UV light (254 and 365 nm). Specific derivatization reagents were applied to detect different phytochemical classes, including ferric chloride (2% FeCl_3_), aluminum chloride (5% AlCl_3_), vanillin (1% in ethanol) followed by sulfuric acid (10% in ethanol), and potassium hydroxide (5% KOH). The phytochemical classes were qualitatively identified based on the characteristic color reactions produced after derivatization and comparison with the corresponding reference standards.

### 4.4 Determination of Total Polyphenols and Tannins

Total polyphenol content (TP) was determined using the Folin–Ciocalteu colorimetric assay with gallic acid as standard [55]. Briefly, aliquots of the extract were reacted with Folin–Ciocalteu reagent and sodium carbonate solution, and absorbance was measured at 760 nm.

Total tannins (TT) were determined by protein precipitation, with tannin content calculated as the difference between total polyphenols and non‐precipitable polyphenols [55]. All measurements were performed in triplicate, and results were expressed as gallic acid equivalents (GAE). Detailed volumes, dilutions, and incubation times are provided in the Supporting Information.

### 4.5 HPLC Analysis of Chemical Markers

The chemical profile of GTE was analyzed by high‐performance liquid chromatography (HPLC). Analyses were carried out on LC‐2060C 3D (Shimadzu Corporation, Kyoto, Japan) equipped with a Phenomenex Hyperclone C18 column (250 × 4.6 mm, 5 µm). The mobile phase consisted of solvent A (water containing 0.1% formic acid) and solvent B (acetonitrile containing 0.1% formic acid) at a flow rate of 0.5 mL·min^−^^1^. The gradient program was: 0–5 min, 10% B; 5–35 min, 10%–40% B; and 35–40 min, 10% B. The column temperature was maintained at 45°C and the injection volume was 10 µL. Detection was performed using a UV detector scanning from 190 to 800 nm, with quantification at 254 nm. Compounds were identified by comparison of retention times with reference standards (caffeine, gallic acid, rutin, and EGCG) and confirmed by co‐injection. Quantification was performed using calibration curves constructed with caffeine (0.5–25 µg·mL^−^^1^).

### 4.6 Pesticide Residue Analysis

Pesticide residues in the plant material were evaluated using a modified QuEChERS method and the QuPPe method [42]. Analyses were performed by liquid chromatography–tandem mass spectrometry (LC–MS/MS) and gas chromatography–tandem mass spectrometry (GC–MS/MS). LC–MS/MS analyses were carried out using an Agilent 6495 triple quadrupole mass spectrometer (Agilent Technologies, Santa Clara, CA, USA), whereas GC–MS/MS analyses were performed on a Thermo TSQ Quantum GC system (Thermo Fisher Scientific, Waltham, MA, USA). Sample preparation and extraction procedures followed previously reported protocols. Detailed chromatographic conditions, gradients, and instrumental parameters are provided in the Supporting Information.

### 4.7 Preparation of Green Tea Extract‐Loaded Nanoparticles

Lipid–polymeric nanoparticles containing GTE were prepared by a solvent emulsification–evaporation method. Briefly, GTE (2 mg) was first dispersed in an aqueous phase containing Tween 20. A second aqueous phase containing Kolliphor P188 was prepared separately. The organic phase consisted of dichloromethane and acetone containing PCL and phosphatidylcholine (Lipoid S100). The extract‐containing aqueous phase was added to the organic phase under an ice bath and emulsified using an Ultra‐Turrax T25 homogenizer (IKA‐Werke GmbH & Co. KG, Staufen, Germany) at 16,000 rpm. The second aqueous phase was subsequently added and the system was further emulsified. The resulting emulsion was magnetically stirred for 24 h to allow complete evaporation of organic solvents. Blank nanoparticles were prepared following the same procedure without addition of the extract.

### 4.8 Physicochemical Characterization of Nanoparticles

Particle size (nm), zeta potential (mV)l and polydispersity index (PdI) of the nanoparticles were determined by dynamic light scattering (DLS) using Zetasizer Nano Series (Malvern Panalytical Ltd., Malvern, Worcestershire, UK). Measurements were performed at predetermined time intervals (1, 7, 14, 21, 28, 60, and 90 days) to evaluate colloidal stability during storage.

Encapsulation efficiency (EE%) was determined by HPLC using caffeine as a chemical marker. Total caffeine was quantified after nanoparticle disruption, whereas non‐encapsulated caffeine was determined in the external aqueous phase. Encapsulation efficiency was calculated according to the following equation:

$$\mathrm{EE}\;\left(\%\right)=100\;\times \;\frac{\mathrm{peak}\;\mathrm{area}\;\mathrm{free}\;\mathrm{caffeine}\;\;}{\mathrm{peak}\;\mathrm{area}\;\mathrm{total}\;\mathrm{caffeine}}$$

### 4.9 Cytotoxicity Assay in ARPE‐19 Cells

ARPE‐19 cells were cultured in DMEM/F12 medium supplemented with 10% fetal bovine serum and maintained at 37°C in a humidified atmosphere containing 5% CO_2_. Cells were seeded in 96‐well plates at a density of 5 × 10^5^ cells per well. After 24 h, the culture medium was replaced with fresh medium containing 2% FBS and different concentrations of GTE (0.05–0.7 mg mL^−^^1^). Cells were incubated for 24, 48, or 72 h. Cell viability was determined using the MTT assay according to the manufacturer's instructions. Absorbance was measured at 540 nm using a Multiskan Sky spectrophotometer (Thermo Fisher Scientific, Vantaa, Finland). Viability percentages were calculated relative to untreated controls. Half‐maximal Inhibitory Concentration (IC_50_) values were determined using a sigmoidal dose–response model.

### 4.10 HET‐CAM Irritation Assay

The HET‐CAM and CAM assays were performed using fertilized chicken eggs before the development of the embryonic nervous system (prior to the stage at which the embryo is considered a live experimental animal under the applicable regulations). Therefore, these procedures did not require approval by an Institutional Animal Care and Use Committee.

The irritation potential of the formulations was evaluated using the hen's egg test on the chorioallantoic membrane (HET‐CAM). Fertilized *Gallus gallus domesticus* eggs were incubated at 37 ± 2°C and approximately 50% relative humidity. On day 10 of embryonic development, 300 µL of each sample were applied directly onto the exposed CAM surface. The tested groups included GTE, blank nanoparticles, GTE‐loaded nanoparticles, sodium hydroxide solution (0.1 mol L^−^^1^, positive control), and saline solution (negative control).

Membrane responses (hemorrhage, lysis, and coagulation) were monitored for 300 s, and the irritation score (IS) was calculated according to the standard formula described in the German HET‐CAM protocol [33].

### 4.11 CAM Antiangiogenic Assay

Antiangiogenic activity was evaluated using the chicken chorioallantoic membrane (CAM) assay [56]. Fertilized eggs were incubated at 37°C with 50%–60% relative humidity. On day 3 of development, albumen removal was performed to detach the embryo from the shell membrane. On day 5, 50 µL of each treatment were applied onto the CAM surface (*n* = 10 per group). The experimental groups included GTE, blank nanoparticles, GTE‐nanoparticle, PBS (negative control), and bevacizumab (6.25 mg mL^−^^1^ in PBS) as positive control. Eggs were further incubated until day 7, after which CAMs were exposed and imaged using a stereomicroscope coupled to a digital camera. Images were acquired at 20x magnification and vascularization was quantified using ImageJ software.

### 4.12 Statistical Analysis

Statistical analyses were performed using RStudio (version 2025.05.0‐496). Data normality was evaluated using the Shapiro–Wilk test. Differences between experimental groups and the negative control were assessed using the Kruskal–Wallis test followed by Dunn's post hoc test. Values of *p* < 0.05 were considered statistically significant.

For cell viability assays, statistical analyses were performed using SigmaPlot 10.0 (Systat Software). Data are expressed as mean ± SEM, and comparisons were performed using Student's t‐test with significance set at *p* < 0.05.

## Author Contributions

**Sarah Kelly Fernandes** contributed to investigation, data curation, visualization, and writing – original draft. **Anízio Bastos de Souza** contributed to investigation, validation, and writing – original draft. **Líbia Andressa Leandro de Paula** contributed to investigation and visualization. **Alessandra Cristine de Souza Matavel** contributed to methodology, investigation, formal analysis, and resources. **Marcos Paulo Gomes Mol** contributed to formal analysis and visualization. **Mariana de Oliveira Almeida** contributed to investigation, validation, and resources. **Vanessa Heloísa Ferreira de Faria** contributed to investigation and validation. **Carolina Paula de Souza Moreira** contributed to conceptualization, supervision, project administration, funding acquisition, and writing – review and editing. All authors have given approval to the final version of the manuscript.

## Conflicts of Interest

The author(s) declared that this work was conducted in the absence of any commercial or financial relationships that could be construed as a potential conflicts of interest.

## Generative AI Statement

During the preparation of this manuscript, the authors used ChatGPT (OpenAI) exclusively to improve the English language, enhance readability, and assist in restructuring sections of the text. No AI‐generated scientific content, data, analyses, figures, or conclusions were used. All scientific interpretations, experimental results, and final manuscript content were critically reviewed, verified, and approved by the authors, who take full responsibility for the published work.

## Supporting information




**Supporting File**: cbdv71893‐sup‐0001‐SuppMat.pdf

## Data Availability

The data that support the findings of this study are available from the corresponding author upon reasonable request.
