# Supplementary material for: Lipid–Polymeric Nanoencapsulation of Standardized Camellia sinensis Extract Enhances Antiangiogenic Activity and Ocular Biocompatibility
Source: Chem Biodivers. 2026 Nov 1;23(11):e71893. doi: 10.1002/cbdv.71893 (PMC13724159; doi:10.1002/cbdv.71893)
Supplement: Supplementary file 1 [file CBDV-23-e71893-s001.pdf]

SUPPORT INFORMATION

1. **Quantification of Total Polyphenols and Tannins**

Total polyphenol (TP) and total tannin (TT) contents in the *Camellia sinensis* extract were determined using colorimetric assays based on the Folin–Ciocalteu method, with gallic acid as the reference standard.

Total polyphenols were quantified by reacting appropriately diluted extract solutions with Folin–Ciocalteu reagent, followed by alkalinization with sodium carbonate. After incubation at room temperature, absorbance was measured at 760 nm, and TP content was calculated from a gallic acid calibration curve (0.1–2.0 $mg{\cdot}mL^{-1}$). Results were expressed as gallic acid equivalents.

Total tannins were determined using an indirect protein precipitation method. Briefly, total polyphenols (A_1) were first measured as described above. Non-precipitable polyphenols (A_2) were then quantified after treatment of the extract with hide powder, followed by filtration and reaction with Folin–Ciocalteu reagent. The tannin content was calculated from the difference between total and non-precipitable polyphenols, normalized to a gallic acid reference (A_3), according to Equation S1.

$$\text{TT (\%)} = 100 \text{ x } \frac{(A_1 - A_2).m_2}{A_3 \,.m_1}$$

where A_1 is the absorbance of total polyphenols, A_2 is the absorbance of non-precipitable polyphenols, A_3 is the absorbance of the gallic acid standard, m_1 is the mass of extract, and m_2 is the mass of standard.

All measurements were performed in triplicate and reported as mean ± standard deviation.

2. Pesticide Residue Analysis

Pesticide residues in the Camellia sinensis extract were evaluated using two complementary validated analytical approaches: (i) a multiresidue method based on a modified QuEChERS EN protocol and (ii) a polar pesticide method based on a modified QuPPE-PO protocol. Analyses were performed using LC–MS/MS and GC–MS/MS following internationally accepted principles for pesticide residue analysis.

2.1. Multiresidue Pesticide Analysis (Modified QuEChERS EN Method)

Sample Preparation and Clean-Up: Approximately 10.0 g of dried extract were weighed into a 50 mL polypropylene centrifuge tube, followed by the addition of 10 mL of acetonitrile containing 1% (v/v) acetic acid. The mixture was shaken for 1 min, after which a salt mixture ($MgSO_4$ and sodium acetate) was added. The tube was immediately shaken for 1 min and centrifuged at 4000 rpm for 5 min at room temperature.

An aliquot (6.0 mL) of the supernatant was subjected to dispersive solid-phase extraction (d-SPE) using $MgSO_4$ and primary secondary amine (PSA), vortexed for 30 s, centrifuged at 4000 rpm for 5 min, and filtered through a 0.22 µm PTFE membrane prior to analysis.

Instrumental Analysis: LC–MS/MS analyses were performed on an Agilent 6495 triple quadrupole system equipped with a C18 reversed-phase column, using water and methanol mobile phases containing ammonium formate and formic acid at a flow rate of 0.6 $mL \cdot min^{-1}$. GC–MS/MS analyses were conducted on a triple quadrupole system equipped with a 5%-phenyl–methylpolysiloxane capillary column, using helium as the carrier gas at 1.2 $mL \cdot min^{-1}$ under temperature-programmed conditions.

Identification and Quantification Criteria: Pesticides were identified based on retention time matching and at least two MRM transitions per compound. Quantification was performed using matrix-matched calibration curves. Limits of quantification (LOQ) were established during method validation. Only carbendazim was detected by LC–MS/MS at concentrations below the LOQ, and no additional residues were observed.

2.2. Polar Pesticide Analysis (Modified QuPPE-PO Method)

Polar pesticide residues were evaluated by extracting 5.0 g of dried extract with 10 mL of acidified methanol. The mixture was shaken for 1 min, centrifuged at 4000 rpm for 5 min, and the supernatant was filtered through a 0.22 µm membrane prior to analysis.

Analyses were performed by LC–MS/MS using a HILIC anionic polar pesticides column under gradient elution at a flow rate of 0.5 $mL \cdot min^{-1}$, with detection in MRM mode. No polar pesticide residues were detected above the established limits of quantification.

Table of pesticides analyzed in green tea.

#	**Pesticide**	**Analytical Technique**
1	Acefate	LC
2	Acetamiprid	LC
3	Alachlor	GC
4	Aldrin	GC
5	Ametoctradin	LC
6	Ametryn	LC and GC
7	Aminocarb	LC
8	Atrazine	LC and GC
9	Deethylatrazine (DEA)	LC
10	Deisopropylatrazine (DIA)	LC
11	Azaconazole	LC and GC
12	Azoxystrobin	LC and GC
13	Benalaxyl	LC
14	Benfuracarb	LC
15	Emamectin benzoate	LC
16	Bifenthrin	GC
17	Bromophos	GC

18	Bromopropylate	GC
19	Boscalid	LC
20	Bupirimate	LC
21	Buprofezin	LC
22	Piperonyl butoxide	LC
23	Cadusafos	LC
24	Carbendazim	LC
25	Carbophenothion	GC
26	Carbofuran	LC and GC
27	3-OH-Carbofuran	LC
28	Carboxin	LC
29	Cyantraniliprole	LC
30	Cyazofamid	LC
31	Cyfluthrin	GC
32	Cymoxanil	LC
33	Cypermethrin	GC
34	Cyproconazole	GC
35	Cyprodinil	LC
36	Cyromazine	LC
37	Clethodim	LC
38	Clofentezine	LC
39	Clomazone	LC
40	Chlorantraniliprole	LC
41	Chlordane gamma	GC
42	Chlorfenvinphos	LC and GC
43	Chlorfluazuron	LC
44	Chlormequat	LC
45	Chlorothalonil	GC
46	Chlorpyrifos	LC and GC
47	Chlorpyrifos ethyl	GC
48	Chlorpyrifos methyl	GC
49	Chlorpyrifos oxon	LC
50	Chlorpropham	GC
51	Chlorthiophos	GC
52	Coumaphos	LC
53	DDD 2,4	GC
54	DDD 4,4	GC
55	DDE 2,4	GC
56	DDE 4,4	GC
57	DDT 2,4	GC
58	DDT 4,4	GC
59	Deltamethrin	LC and GC
60	Diazinon	LC
61	Dichlofluanid	GC
62	Dichloran	GC

63	Dicrotophos	LC
64	Dieldrin	GC
65	Diethofencarb	LC
66	Difenoconazole	GC
67	Dimethoate	LC
68	Dimethomorph	LC
69	Dinotefuran	LC
70	Disulfoton	GC
71	Disulfoton sulfoxide	GC
72	Diuron	GC
73	Dodemorph	GC
74	Endosulfan alpha	LC
75	Endosulfan beta	LC
76	Endosulfan sulfate	LC
77	Endrin	LC
78	Spinosad	LC
79	Spinetoram	LC
80	Spiroxamine	LC
81	Ethiofencarb	LC
82	Ethiofencarb sulfone	LC
83	Ethiofencarb sulfoxide	LC
84	Ethion	LC
85	Etoprophos	LC
86	Etrimfos	LC
87	Fenbuconazole	LC
88	Fenpyroximate	LC
89	Fenpropathrin	LC
90	Fenpropidin	LC
91	Fenpropimorph	LC
92	Fenamiphos	LC
93	Fenamiphos sulfoxide	LC
94	Fenarimol	LC
95	Fenitrothion	LC
96	Fenpropidin	LC
97	Fenthion	LC
98	Fenthion oxon	LC
99	Fenthion sulfoxide	LC
100	Fipronil	LC
101	Flonicamid	LC
102	Fluorpyrauxifen benzyl	LC
103	Fluazinam	LC
104	Flubendiamide	LC
105	Flufenoxuron	LC
106	Flumetralin	LC
107	Fluquinconazole	LC

108	Flutolanil	LC
109	Flutriafol	LC
110	Fluxapyroxad	LC
111	Fomesafen	LC
112	Formetanate (Hydroxide)	LC
113	Phosalone	LC
114	Phosphamidon	LC
115	Fosthiazate	LC
116	Furathiocarb	LC
117	Haloxyfop-p-methyl	LC
118	HCH alpha	LC
119	HCH beta	LC
120	HCH delta	LC
121	HCH gamma (Lindane)	LC
122	Heptachlor	LC
123	Heptachlor endo-epoxide	LC
124	Heptachlor exo-epoxide	LC
125	Heptenophos	LC
126	Hexachlorobenzene (HCB)	LC
127	Hexaconazole	LC
128	Hexazinone	LC
129	Imidacloprid	LC
130	Indoxacarb	LC
131	Lactofen	LC
132	Linuron	LC
133	Lambda-cyhalothrin	LC
134	Malathion	LC
135	Mandipropamid	LC
136	Metalaxyl	LC
137	Methamidophos	LC
138	Methiocarb	LC
139	Metolachlor	LC
140	Methoxychlor	LC
141	Methoxyfenozide	LC
142	Metribuzin	LC
143	Mevinphos	LC
144	Mirex	LC
145	Molinate	LC
146	Monocrotophos	LC
147	Nitenpyram	LC
148	Nuarmilol	LC
149	Omethoate	LC
150	Oxadixyl	LC
151	Oxamyl	LC
152	Oxasulfuron	LC

153	Oxyfluorfen	LC
154	Paclobutrazol	LC
155	Paraoxon-ethyl	LC
156	Parathion-ethyl	LC
157	Parathion-methyl	LC
158	Pencycuron	LC
159	Pendimethalin	LC
160	Permethrin	LC
161	Picoxystrobin	LC
162	Piperonyl butoxide	LC
163	Pyraclofos	LC
164	Pyridafenthion	LC
165	Pyrimethanil	LC
166	Pirimicarb	LC
167	Pirimiphos-ethyl	LC
168	Pirimiphos-methyl	LC
169	Pyriproxyfen	LC
170	Procymidone	LC
171	Profenofos	LC
172	Profoxydim	LC
173	Prometryn	LC
174	Propamocarb	LC
175	Propanil	LC
176	Propoxur	LC
177	Prothiofos	LC
178	Quinalphos	LC
179	Quintozene	LC
180	Quizalofop-p-ethyl	LC
181	Rotenone	LC
182	Simazine	LC
183	Sulfentrazone	LC
184	Sulfluramid	LC
185	Sulfometuron-methyl	LC
186	Sulphotep	LC
187	Tebuconazole	LC
188	Tebuthiuron	LC
189	Terbufos	LC
190	Terbuthylazine	LC
191	Tetraconazole	LC
192	Tetradifon	LC
193	Thiabendazole	LC
194	Thiacloprid	LC
195	Thiamethoxam	LC
196	Thiobencarb	LC
197	Tolylfluanid	LC

198	Triazophos	LC
199	Tricyclazole	LC
200	Trifluralin	LC
201	Vamidothion	LC
202	Vinclozolin	LC
203	Chlormequat	LC
204	Ethephon	LC
205	Fosfomycin	LC
206	Glufosinate	LC
207	Mepiquat	LC

3. ImageJ-Based Quantification of Vascularized Area in the CAM Model after Treatment

sample	area
PBS_1	49.373
PBS_2	49.648
PBS_3	51.653
PBS_4	51.653
PBS_5	52.478
PBS_6	56.601
PBS_7	56.637
PBS_8	59.043
PBS_9	59.043
PBS_10	59.971
BVZ_1	28.505
BVZ_2	31.498
BVZ_3	32.165
BVZ_4	32.388
BVZ_5	32.811
BVZ_6	33.170
BVZ_7	33.815
BVZ_8	34.321
BVZ_9	34.383
BVZ_10	34.662
GTE_1	28.590
GTE_2	28.292
GTE_3	28.386
GTE_4	28.529
GTE_5	29.599
GTE_6	29.949
GTE_7	34.430
GTE_8	36.499
GTE_9	36.811

GTE_10	38.439
B-NP_1	47.848
B-NP_2	50.687
B-NP_3	51.937
B-NP_4	53.024
B-NP_5	53.554
B-NP_6	54.452
B-NP_7	55.369
B-NP_8	55.682
B-NP_9	56.311
B-NP_10	56.746
GTE-NP_1	36.688
GTE-NP_2	38.852
GTE-NP_3	40.170
GTE-NP_4	40.965
GTE-NP_5	41.898
GTE-NP_6	42.965
GTE-NP_7	43.232
GTE-NP_8	44.324
GTE-NP_9	44.762
GTE-NP_10	44.808
